# A Sensitive Spectrophotometric Method for the Determination of Pregabalin in Bulk, Pharmaceutical Formulations and in Human Urine Samples

**Published:** 2009-12

**Authors:** Rajinder Singh Gujral, Sk Manirul Haque, Prem Shanker

**Affiliations:** *Vardhman Chemtech Ltd, Nimbua, Dera Bassi, Mohali (Punjab) India*

**Keywords:** pregabalin, validation, bulk drug, pharmaceutical formulations, human urine samples

## Abstract

A simple and sensitive spectrophotometric method was developed and validated for the determination of pregabalin in bulk, pharmaceutical formulations and in human urine samples. The method was based on the reaction of drug with the mixture of potassium iodate and potassium iodide. The method was linear in the range of 0.5–3.5 μg/ml. There is no official method for the determination of pregabalin. The absorbance was measured at 353 nm. The method was validated with respect to accuracy, precision, specificity, ruggedness, robustness, limit of detection and limit of quantitation. This method was used successfully for the quality assessment of five pregabalin drug products and in human urine samples with good precision and accuracy.

## INTRODUCTION

**Figure 1:**
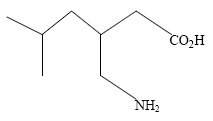
Structure of Pregabalin.

Pregabalin [S-[+]-3-isobutyl GABA or (S)-3-(amino methyl)-5-methylhexanoic acid, Lyrica] (Fig. 1) is an anticonvulsant and analgesic medication that is both structurally and pharmacologically related to gabapentin (Neurotonin; Pfizer Inc; New York, NY). It was recently approved for adjunctive treatment of partial seizures in adults (1–4) in both the United States and Europe and for the treatment of neuropathic pain from postherpetic neuralgia and diabetic neuropathy. The compound was originally synthesized with the hope of modulating brain GABA receptors or GABA synthetic enzymes. These compounds are inactive at GABA_A_ and GABA_B_ receptors. The mechanism of action of Pregabalin has been characterized only partially, and in particular, the cellular and molecular details of its action to reduce neurotransmitter release are incompletely known.

Pregabalin undergoes minimal metabolism in human with unchanged parent representing the majority (≥90 %) of drug-derived material (5). This contrasts with gabapentin, which is absorbed via a capacity limited L-amino acid transport system from the proximal small bowel into the blood stream (6, 7).

The therapeutic importance of Pregabalin was behind the development of numerous methods for its determination. The methods adapted to the analysis of PGB include high–performance liquid chromatography (HPLC) (8), liquid chromatography-mass spectrophotometry (LC-MS) (9, 10) and spectrofluorimetry (11). The determination in biological fluids normally requires the use of trace analysis techniques such as HPLC, LC, capillary electrophoresis (CE), cyclic voltametry, LC-MS, gas chromatography – mass spectrophotometry (GC-MS), inductively coupled plasma – mass spectrophotometry. These methods require long and tedious pretreatment of the samples and laborious clean up procedures prior to analysis. An official monograph of PGB does not exist in any pharmacopoeia and determination of PGB in bulk and pharmaceutical formulations has not been yet described. Therefore, it is very imperative to develop a simple and suitable analytical method for the determination of PGB in bulk and pharmaceutical formulations. UV-Visible spectrophotometry is the technique of choice in research laboratories, hospitals and pharmaceutical industries due to its low cost and inherent simplicity.

This paper reports a simple, sensitive and accurate spectrophotometric method for the determination of PGB. The method was based on the reaction of drug with the mixture of potassium iodide and potassium iodate and absorbance was measured at 353 nm. The proposed method was extended to the determination of PGB in bulk, pharmaceutical formulations and in human urine samples.

## EXPERIMENTAL

### Apparatus

Spectral runs were made on UV 3000^+^ UV/VIS spectrophotometer (LABINDIA^®^, Mumbai, India) with 1 cm matched glass cell.

### Materials and Reagents

- Pregabalin (Vardhman Chemtech Ltd, Punjab, India) was used as working standard;
- Pharmaceutical formulations of PGB such as Gabanext 75 (Nicholas Piramal India Ltd, Mumbai, India), Pregalin 75 (Torrent Pharmaceutical Ltd, Baddi, India), Neugaba 75 (Sun Pharmaceutical Industries, Jammu, India), Mahagaba 75 (Mankind Pharma Ltd, New Delhi, India) and Maxgalin 75 (Sun Pharmaceutical Industries, Jammu, India) were purchased from local markets;
- Potassium iodate (KIO_3_) was purchased from RFCL Limited (New Delhi, India);
- Potassium iodide (KI) was purchased from RFCL Limited (New Delhi, India);
- Sodium carbonate was purchased from Qualigens fine chemicals (Mumbai, India);
- Sodium bicarbonate was purchased from Qualigens fine chemicals (Mumbai, India);
- Urine samples were obtained from healthy volunteers;
- Carbonate buffer of pH 9.4 was prepared by dissolving 26.5 gm sodium carbonate and 21.0 gm sodium bicarbonate in 500 ml distilled water;
- All other chemicals were of analytical grade and used without any further purification.

### Standard PGB Solution

A stock solution of PGB (50 μg/ml) was prepared by dissolving 5 mg PGB in 100 ml volumetric flasks with double distilled water. The stock solution (50 μg/ml) was used to prepare the working solutions by suitable dilutions with distilled water. The solutions were stable at least 10 days in room temperature.

## METHODS

### Procedure for the determination of PGB

**Figure 2:**
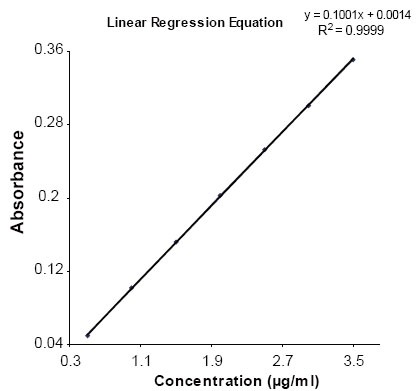
Linear Regression equation of the proposed method.

Aliquots of stock solution (50 μg/ml) were pipetted into a series of 10 ml volumetric. To each flask, 1.0 ml 4 × 10^−2^ M KI and 2.5 ml 5 × 10^−3^ M KIO_3_ were added and diluted to volume with distilled water. The reaction was allowed to proceed at room temperature. The calibration curve was constructed by plotting absorbance against the initial concentration of PGB. The linearity range or Beer’s range follows in the range between 0.5 to 3.5 μg/ml (Fig. 2). The content of PGB was calculated either from the calibration curve or corresponding regression equation and found that the absorbance is stable for at least ten days at room temperature.

### Procedure for determination of PGB in pharmaceutical formulations

One capsule (claiming 75 mg of Pregabalin) was accurately weighed and finely powdered. A quantity of the powder equivalent to 5 mg of PGB was extracted by shaking with 20 ml of distilled water, followed by another two extractions each with 10 ml distilled water. After passing through a 0.45 μm Millipore filter, the solution was diluted with distilled water to obtain a concentration of about 50 μg/ml. It was further diluted according to the need and then analyzed following the proposed procedures. The nominal content of the capsule was calculated either from the previously plotted calibration graphs or using regression equation.

### Procedure for determination of PGB in human urine samples

Aliquot volumes of human urine samples were transferred into small separating funnel. 10 ml of carbonate buffer pH-9.4 was added and solution was mixed well. The solution was then extracted with 3 × 10 ml diethyl ether. The ether extract was collected and evaporated. The residue was dissolved in 10 ml distilled water and above general procedure was then followed. The amount of PGB was obtained from the calibration graphs or corresponding regression equation.

## RESULTS AND DISCUSION

### Reaction with a mixture of iodide and iodate

It has been reported in the literature (12) that iodine is formed as a result of the interaction of a mixture of iodide and iodate with inorganic or organic acid in accordance with the equation:

$$5\;{\text{I}}^{-}+{\text{IO}}_{3}^{-}+6\;{\text{H}}^{+}\to 3\;{\text{H}}_{2}\text{O}+3\;{\text{I}}_{2}$$

**Figure 3:**
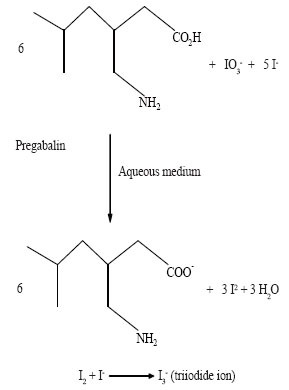
This is reaction mechanism of formation of TriIodide ion.

In aqueous medium, the iodide ions react with the liberated iodine to yield triiodide ion (I_2_ + I^−^ → I_3_^−^) which detected in UV detector at 353 nm. We thought that this reaction would be helpful for developing a spectrophotometric method for determination of PGB as it contains - COOH group in its moiety. Keeping this in mind, a mixture of potassium iodide and iodate was allowed to react with PGB which yielded iodine. Then the liberated iodine reacted with the excess of iodide ion resulting in the formation of triiodide ion (Fig. 3).

### Optimization of reaction conditions

The different parameters affecting the development process were extensively studied to determine the optimum conditions for the assay procedures. The optimum values of the variables were maintained throughout the determination process.

**Figure 4:**
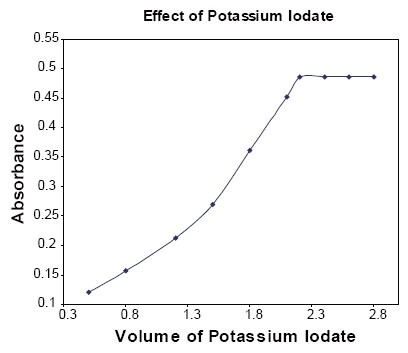
Effect of the volume of KIO_3_ (5.0 × 10^−3^ M); keeping constant 0.5 ml of 50 μg/ml PGB and KI (3.2 × 10^−2^ M).

**Effect of the concentration of potassium iodate:** The effect of the volume of 5 × 10^−3^ M potassium iodate on the absorbance of the product was studied in the range of 0.2–2.8 ml. The absorbance increases with the increase in the volume of potassium iodate and became constant at 2.2 ml. Further addition of KIO_3_ does not change in the absorbance and therefore, 2.5 ml of 5 × 10^−3^ M KIO_3_ was chosen as an optimum value (Fig. 4).

**Figure 5:**
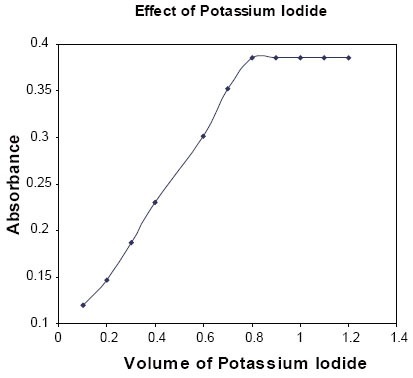
Effect of the volume of KI (4.0 × 10^−2^ M); keeping constant 0.5 ml of 50 μg/ml PGB and KIO_3_ (12.5 × 10^−3^ M).

**Effect of the concentration of potassium iodide:** The effect of the volume 4 × 10^−2^ M potassium iodide on the absorbance of the product was studied in the range of 0.1–1.2 ml, keeping the constant concentrations of PGB (50 μg/ml) and KIO_3_ (12.5 × 10^−3^ M). The maximum absorbance was obtained with 0.8 ml; further addition caused no change on the absorbance. Thus, 1.0 ml of 4 × 10^−2^ M potassium iodide (Fig. 5) was used throughout the experiment.

### Method Validation

The method was validated for selectivity, linearity, precision, accuracy, recovery and stability according to the principles of the Food and Drug Administration (FDA) industry guidance (13). The specificity and selectivity of the proposed method was evaluated by estimating the amount of PGB in the presence of common excipients lactose monohydrate, corn, starch, talc and methyl cobalamin.

The linearity of the proposed method was constructed for Pregabalin reference standard solution by plotting concentration of the compound versus the absorbance. The linearity was evaluated by linear regression analysis, which was calculated by the least square regression method. The parameters LOD and LOQ were determined on the basis of response and slope of the regression equation. The accuracy and precision of the method was evaluated within the linear range based on the analysis of PGB reference standard samples and pharmaceutical formulations at 1.0, 2.0 and 3.0 μg/ml. Five independent analysis were performed at each concentrations level within one day (intraday precision) as well as for five consecutive days (interday precision). The accuracy was ascertained by recovery studies using the standard addition method. The proposed method was used for estimation of PGB from capsules after spiking with 50, 150 and 250 % additional pure drug. The amount of PGB was determined from the regression equation.

The absorbance-concentration plot for the proposed method was found to be rectilinear over the range of 0.5–3.5 μg/ml. Linear regression analysis of calibration data gave the regression equation cited in Table 1 with correlation coefficients close to unity. Statistical analysis of regression lines were made regarding the standard deviation of residuals (S_x/y_), standard deviation of slopes (S_b_) and standard deviation of intercepts (S_a_) and the values are summarized in Table 1.

**Table 1:** Summary of optical and regression characteristics of the proposed method

Parameters	Pregabalin

Linear dynamic range (μg/ml)	0.50–3.50
Regression equation^a^	Y = 1.001 × 10^−1^ *X* + 1.4 × 10^−3^
S_a_	9.13 × 10^−4^
t S_a_ b	2.03 × 10^−3^
S_b_	2.60 × 10^−4^
t S_b_ b	5.79 × 10^−4^
Correlation coefficient (r)	0.9999
LOD (μg/ml)	2.46 × 10^−1^
LOQ (μg/ml)	8.154 × 10^−2^
Variance (S_o_^2^) of calibration line	1.53 × 10^−6^

a With respect to Y = a + b *X*, where X is the concentration in μg/ml, Y is Absorbance;

b Confidence interval of the intercept and slope at 95 % confidence level and ten degrees of freedom (t=2.228)

The within day precision assays were carried out through replicate analysis (n=5) of PGB corresponding to 1.0, 2.0 and 3.0 μg/ml. The interday precision was evaluated through replicate analysis of the pure drug samples for five consecutive days at the same concentration levels as used in within day precision. The results of these assays are reported in Table 2. As can be seen from Table 2 that the recovery and RSD values for within day precision were always lower than 100.10% and 0.799%; recovery and RSD values for interday precision were lower than 100.07% and 1.001%. The precision results are satisfactory. The intraday and interday precision assays were also carried for PGB in pharmaceutical formulations. The results are summarized in Table 3. As can be seen from Table 3 that the recovery and RSD values were in the ranges 99.85 to 100.07%; 0.133 to 1.20% and 99.90 to 100.10%; 0.167 to 1.001% respectively for intraday and interday precision.

**Table 2:** Summary of accuracy and precision results of the proposed method in pure form.

Proposed methods	Amount (μg/ml)		RSD	REC.	SAE^b^	C.L.^c^
	Taken	Found ± SD^a^				

Intra day assay	1.00	1.001 ± 0.008	0.799	100.10	3.6 × 10^−3^	1.0 × 10^−2^
	2.00	1.999 ± 0.006	0.300	99.950	2.7 × 10^−3^	7.5 × 10^−3^
	3.00	3.001 ± 0.004	0.133	100.03	1.8 × 10^−3^	5.0 × 10^−3^
Inter day assay	1.00	0.9999 ± 0.012	1.200	99.99	5.4 × 10^−3^	1.5 × 10^−2^
	2.00	2.001 ± 0.009	0.450	100.05	4.0 × 10^−3^	1.1 × 10^−2^
	3.00	3.002 ± 0.006	0.200	100.07	2.7 × 10^−3^	7.5 × 10^−3^

a Mean for 5 independent analyses;

b SAE, standard analytical error;

c C.L., confidence limit at 95 % confidence level and 4 degrees of freedom (t=2.776).

**Table 3:** Summary of accuracy and precision results of the proposed method in pharmaceutical formulations

Proposed methods	Amount (μg/ml)		RSD	REC.	SAE^b^	C.L.^c^
	Taken	Found ± SD^a^				

Intra day assay						
Gabanext-75	1.00	1.000 ± 0.010	1.000	100.000	0.0045	0.0124
Gabanext-75	2.00	1.999 ± 0.006	0.300	99.950	0.0027	0.0075
Gabanext-75	3.00	3.001 ± 0.004	0.133	100.03	0.0018	0.0050
Pregalin-75	1.00	0.999 ± 0.012	1.200	99.90	0.0054	0.0015
Pregalin-75	2.00	2.001 ± 0.009	0.450	99.950	0.0040	0.0011
Pregalin-75	3.00	3.002 ± 0.006	0.200	100.07	0.0027	0.0075
Neugaba-75	1.00	0.999 ± 0.011	1.101	99.90	0.0049	0.0137
Neugaba-75	2.00	1.998 ± 0.021	1.051	99.90	0.0094	0.0261
Neugaba-75	3.00	3.002 ± 0.013	0.433	100.07	0.0058	0.0161
Maxgalin-75	1.00	1.000 ± 0.012	1.200	100.00	0.0054	0.0015
Maxgalin-75	2.00	2.001 ± 0.009	0.450	99.950	0.0040	0.0011
Maxgalin-75	3.00	2.999 ± 0.015	0.500	99.967	0.0067	0.0186
Mahagaba-75	1.00	0.999 ± 0.007	0.700	99.90	0.0031	0.0087
Mahagaba-75	2.00	1.997 ± 0.008	0.401	99.85	0.0036	0.0099
Mahagaba-75	3.00	3.000 ± 0.004	0.133	100.000	0.0018	0.0050
Inter day assay						
Gabanext-75	1.00	1.001 ± 0.002	0.200	100.10	0.0010	0.0025
Gabanext-75	2.00	1.998 ± 0.007	0.350	99.900	0.0031	0.0087
Gabanext-75	3.00	3.002 ± 0.005	0.167	100.07	0.0022	0.0062
Pregalin-75	1.00	0.999 ± 0.010	1.001	99.90	0.0045	0.0124
Pregalin-75	2.00	2.001 ± 0.013	0.650	100.05	0.0058	0.0161
Pregalin-75	3.00	3.000 ± 0.008	0.267	100.00	0.0036	0.0099
Neugaba-75	1.00	1.000 ± 0.009	0.900	100.00	0.0040	0.0111
Neugaba-75	2.00	1.999 ± 0.014	0.700	99.950	0.0063	0.0174
Neugaba-75	3.00	3.001 ± 0.006	0.200	100.03	0.0027	0.0075
Maxgalin-75	1.00	1.000 ± 0.004	0.400	100.00	0.0018	0.0050
Maxgalin-75	2.00	2.000 ± 0.011	0.550	100.00	0.0049	0.0136
Maxgalin-75	3.00	2.999 ± 0.014	0.467	99.967	0.0063	0.0174
Mahagaba-75	1.00	0.999 ± 0.009	0.901	99.90	0.0040	0.0112
Mahagaba-75	2.00	1.998 ± 0.010	0.501	99.90	0.0045	0.0124
Mahagaba-75	3.00	2.999 ± 0.013	0.433	99.967	0.0058	0.0161

a Mean for 5 independent analyses;

b SAE, standard analytical error;

c C.L., confidence limit at 95% confidence level and 4 degrees of freedom (t=2.776).

The proposed method was used for estimating of PGB from capsules after spiking with 50, 150 and 250 % of additional pure drug. The results are reported in Table 4. As can be seen from Table 4 that the recovery and RSD values were in the ranges 99.867 to 100.067 % and 0.200 to 0.800 %. The selectivity of the proposed method was ascertained by analyzing standard PGB in the presence capsule such as lactose monohydrate, corn, starch, talc and methyl cobalamin. It was observed that the excipients did not interfere with the proposed method.

**Table 4:** Summary of data for the determination of pregabalin in pharmaceutical preparations by standard addition method

Formulations	Amount (μg/ml)			Recovery (%)	RSD (%)	SAE^b^
	Taken	Added	Found ± SD^a^			

Capsules						
Gabanext-75	1.00	0.50	1.499 ± 0.009	99.933	0.600	0.0040
	1.00	1.50	2.501 ± 0.010	100.04	0.400	0.0045
	1.00	2.50	3.499 ± 0.011	99.971	0.314	0.0049
Neugaba-75	1.00	0.50	1.498 ± 0.006	99.867	0.401	0.0027
	1.00	1.50	2.500 ± 0.008	100.00	0.320	0.0036
	1.00	2.50	3.498 ± 0.010	99.943	0.286	0.0045
Maxgalin-75	1.00	0.50	1.500 ± 0.012	100.00	0.800	0.0054
	1.00	1.50	2.499 ± 0.013	99.960	0.520	0.0058
	1.00	2.50	3.498 ± 0.009	99.943	0.257	0.0040
Pregalin- 75	1.00	0.50	1.501 ± 0.006	100.067	0.400	0.0027
	1.00	1.50	2.498 ± 0.008	99.920	0.320	0.0036
	1.00	2.50	3.500 ± 0.007	100.00	0.200	0.0031
Mahagaba- 75	1.00	0.50	1.500 ± 0.004	100.00	0.267	0.0018
	1.00	1.50	2.501 ± 0.006	100.04	0.240	0.0027
	1.00	2.50	3.501 ± 0.008	100.03	0.229	0.0036

a Mean for 5 independent analyses;

b SAE, standard analytical error.

The proposed method was further extended to the *in vitro* determination of PGB in spiked human urine samples. In neuropathic patients, PGB is orally given in doses of 150 to 600 mg per day, with an associated mean of around 123 μg.hr/ml. Pregabalin undergoes minimal metabolism in human with unchanged parent representing ≥ 90% of drug derived in urine. This concentration fell well in within working range of proposed method. The calibration graphs were constructed by plotting absorbance versus increasing concentrations of PGB in spiked human urine samples over the concentration range 0.5–3.5 μg/ml. The results (Table 5) are satisfactorily accurate and precise. The performance of the proposed method was studied with other existing method (14). In case of proposed method and reported method, the reported method has higher LOD and LOQ value than the proposed method and summarized in Table 6.

**Table 5:** Application of the proposed spectrophotometric method to the determination of pregabalin in human urine samples

**Amount added (μg/ml)**	**Amount found (μg/ml)**	**Recovery (%)**

1.00	0.9821	98.21
1.50	1.4781	98.54
2.00	1.9741	98.71
2.50	2.4812	99.25
3.00	2.9960	99.87
X		98.92
RSD		0.660

**Table 6:** Comparable table for the proposed method and reported method (14)

Parameters	Proposed Method	Reported Method

Linear dynamic range (μg/ml)	0.50–3.50	0.75–6.00
S_a_	9.13 × 10^–4^	10.06
t S_a_^b^	2.03 × 10^–3^	22.41
S_b_	2.60 × 10^–4^	2.71
t S_b_^b^	5.79 × 10^–4^	6.04
LOD (μg/ml)	2.46 × 10^–1^	0.669
LOQ (μg/ml)	8.154 × 10^–2^	0.221

## CONCLUSIONS

The proposed method does not require any laborious clean up procedure before measurement. In addition, the method has wider linear dynamic range with good accuracy and precision. The method shows no interference from the common excipients and additives. This may help in analyzing affectivity of this drug in human beings during treatment. Therefore, it is concluded that the proposed method is simple, sensitive and rapid for the determination of PGB in bulk, pharmaceutical formulations and in human urine samples.
